# Changes in Quality of *Carya illinoinensis* at Different Harvest Periods

**DOI:** 10.3390/foods13162553

**Published:** 2024-08-16

**Authors:** Xinchen Jiang, Li Cui, Qiuqin Zhang, Tao Zhang, Yaming Qian, Hongmei Xiao, Haijun Zhu

**Affiliations:** 1 Institute of Pomology, Jiangsu Academy of Agricultural Sciences/Jiangsu Key Laboratory for Horticultural Crop Genetic Improvement, Nanjing 210014, China; jxc417565835@163.com (X.J.); 20210103@jaas.ac.cn (T.Z.); qchairman@163.com (Y.Q.); 2 Institute of Agro-Product Processing, Jiangsu Academy of Agricultural Sciences, Nanjing 210014, China; clisu1@163.com; 3 Sanya Institute of Nanjing Agricultural University, Sanya 572024, China; zqq@njau.edu.cn (Q.Z.); xhm@njau.edu.cn (H.X.)

**Keywords:** *Carya illinoinensis* (Wangenh.) K. Koch, Pawnee, Wichita, harvesting period

## Abstract

In order to determine the appropriate harvesting period of *Carya illinoinensis* planted in Nanjing city of China, this study analyzed the phenotypic characteristics and inclusions, including single fruit quality, fruit transverse and vertical diameter, kernel rate, water content, color, respiratory strength, crude fat, soluble sugar, soluble protein, and total phenols, of two cultivars ‘Pawnee’ and ‘Wichita’ during September and October, respectively. Results showed that the respiration intensity and *I_AD_* values of pecan fruits decreased as the harvest date was delayed. ‘Pawnee’ fruits exhibited the highest seed kernel fullness, ∆E value, fruit transverse diameter, shape index, kernel yield, crude fat, and total phenolic content in late September and early October, while the quality of ‘Wichita’ fruits reached its peak in late October. The appropriate harvest period is conducive to the material accumulation of *Carya illinoinensis*, which is of great practical significance for improving the commodity value of pecans. The optimal harvesting period for ‘Pawnee’ in Nanjing is from the end of September to the beginning of October, and the optimal harvesting period for ‘Wichita’ is from mid- to late-October to the end of October.

## 1. Introduction

*Carya illinoinensis* (Wangenh.) K. Koch, commonly known as pecan, is a deciduous tree belonging to the *Carya* genus in the Juglandaceae family. It is native to the United States and northern Mexico [1] and is highly valued for its economic, ecological, and versatile uses [2,3,4,5]. Pecan fruit is rich in oils and phenols, which provide cardiovascular protection and antioxidant benefits, among other health effects [6,7,8,9]. Currently, pecans in China are primarily concentrated in the Yangtze River Basin and areas to the south, including Zhejiang, Jiangsu, Yunnan, Anhui, and Jiangxi [10,11]. In China, pecans mature in September and October, and the harvesting period varies in different regions. The ripening time of pecans also varies with cultivars. Table 1 provides a concise overview of the maturation dates of the principal pecan cultivars in various provinces of China, accompanied by a comparative analysis of the environmental characteristics of the different regions [12,13,14,15,16,17,18,19]. Harvesting is a crucial technical aspect of pecan production. The timing of the harvest directly impacts the yield and quality of pecans. As the pecan ripens, the bright green skin gradually changes to a yellowish green. With delayed harvest, the skin becomes wrinkled and develops dark brown spots. The crack at the top of the green skin deepens and enlarges, making the fruit more prone to falling off. Farmers must carefully choose the harvesting period to ensure optimal results. In the actual production of pecans, if harvested too early, the green skin is extremely difficult to peel, the fruit is immature, the seed kernel is small and astringent, and the commercial rate is low. If harvested too late, the fruit enters the aging period, which leads the fruit to fall and crack, and the resistance against disease decreases. Therefore, in order to avoid the phenomenon of early harvesting or late harvesting of pecan fruit, it is beneficial to determine the appropriate harvesting date for actual production.

This research analyzed the phenotypic traits, crude fat, soluble sugar, soluble protein, and total phenols of pecan fruits at different harvesting periods using ‘Pawnee’ and ‘Wichita’, two excellent pecan varieties with good planting performance, quality, and high yield in the Nanjing area of China [20,21,22,23]. The aim was to provide a reference for the optimal harvesting period of pecans in the Nanjing area.

## 2. Materials and Methods

### 2.1. Experimental Site and Plant Material

This study was conducted in a pecan experimental orchard in Nanjing, China (118°37′24.88′′~118°37′46.57′′ E, 32°28′57.41′′~32°28′59.50′′ N, 30 masl). The test site covers an area of approximately 80,000 m^2^ and is located in a region with a northern subtropical monsoon climate, characterized by four distinct seasons and sufficient heat. The average annual temperature is 22 °C, with an annual maximum of 41 °C and a minimum of −5 °C. The annual precipitation is 430 mm. The frost-free period is 237 days. The trial was conducted using the ‘Pawnee’ and ‘Wichita’ pecan cultivars, which were planted at the Long-term Research Base of Pecan Breeding and Cultivation, Jiangsu Academy of Agricultural Sciences, China. The trees of both cultivars are 10 years old, with uniform and well-developed growth. The trial site was equipped with a protective high fence, and the trees were regularly watered and fertilized. The forest is cleared of weeds by mowers, insecticides are sprayed to control aphids and other pests, and traps are placed at regular intervals to remove the *Dichocrocis punctiferalis*. The orchard is primarily fertilized with nitrogen during the months of March and April, with potash applied mainly during the period of fruit expansion, which occurs between July and August. Irrigation is also employed, with watering occurring once a month from July to February. Additionally, pruning of the larger trees is conducted on two occasions, between November and December.

### 2.2. Sampling Methods

The experiment involved selecting three sample trees from each of the two pecan cultivars with uniform and healthy growth. Green and de-greened fruits were distinguished at the time of sampling. The green fruit of pecan is used to describe the fruit that has been picked directly from the tree with an outer layer of green pericarp. In contrast, the de-greened wet fruit is used to describe the nut fruit with the outer layer of green pericarp removed, either artificially or mechanically. In the production process, the de-greened wet fruit can be transformed into pecan products for sale in the market through a series of cleaning, drying, and other treatments. Sampling commenced when the fruit was almost ripe, but before the skin had split, and concluded when the skin had fully split and the fruit was ripe. The ‘Pawnee’ cultivar was sampled five times on September 15, 20, and 25, as well as on September 30 and October 9. The ‘Wichita’ cultivar was sampled on October 9, 14, 19, 24, and 29. Sampling involved collecting 15 healthy and pest-free pecan fruits from the middle and lower parts of the tree in four directions (east, south, west, and north). A total of 45 fruits from a single tree were collected at a time and cryopreserved for laboratory analysis.

### 2.3. Fruit Morphometry

The weight of green and de-greened wet pecans was measured using an electronic balance with one-thousandth accuracy. The transverse diameter of the wet fruit was measured at its widest point using a vernier caliper with an accuracy of 0.01 mm. The vertical diameter of the pecan wet fruit was measured to the widest part of the base with a precision of 0.01 mm. The obtained values were averaged to calculate the fruit shape index using Equation (1). The kernel rate was calculated using Equation (2). To evaluate the quality of the kernels the color, fullness, and intactness of the kernels, were observed according to the ‘LY/T 2703-2016 Quality Grade of Pecan Nuts and Kernels’ standard [24,25].

(1) $$\mathrm{Fruit}\;\mathrm{shape}\;\mathrm{index}=\frac{\mathrm{fruit}\;\mathrm{vertical}\;\mathrm{diameter}}{\mathrm{fruit}\;\mathrm{transverse}\;\mathrm{diameter}}$$

(2) $$\mathrm{Kernel}\;\mathrm{rate}\;\left(\%\right)=\left(\frac{\mathrm{kernel}\;\mathrm{mass}}{\mathrm{nut}\;\mathrm{mass}}\right)\times 100\%$$

### 2.4. Water Content

Fruit water content was determined according to Equation (3).

(3) $$\mathrm{Water}\;\mathrm{content}=\frac{\mathrm{fresh}\;\mathrm{weight}\;\mathrm{of}\;\mathrm{nuts}\;-\;\mathrm{dry}\;\mathrm{weight}\;\mathrm{of}\;\mathrm{nuts}}{\mathrm{fresh}\;\mathrm{weight}\;\mathrm{of}\;\mathrm{nuts}}\times 100\%$$

### 2.5. Pericarp Color and Index of Absorbance Difference

A portable colorimeter HP-2136 was used in the experiment to measure the pericarp color. Ten fruits were randomly selected from each group and measured at three points on the equatorial plane of the fruit. The international color difference standard CIE (L* (lightness), a* (red-green), b* (yellow-blue)) was adopted, and ΔE (total color difference) was calculated from Equation (4).

(4) $$\Delta E={\left[{\left(\Delta L\right)}^{2}+{\left(\Delta a\right)}^{2}+{\left(\Delta b\right)}^{2}\right]}^{\frac{1}{2}}$$

The pericarp’s index of absorbance difference (*I_AD_*) value was determined using a DA-Meter (TR Turonisrl, Forlì, Italy). Measurements were taken at the center points of the four sides of the fruit, and the results were averaged [26,27].

### 2.6. Respiration Intensity

The respiration intensity of the pecan fruits was measured using a CheckMate-9900 gas analyzer. The respiration rate was calculated using Equation (5). Ten fruits of uniform size were placed in a 2 L respiratory tank for each group. The tank was sealed with petroleum jelly, and readings were taken at *A*_0_ after 0.5 h and *A*_1_ after 1.5 h.

(5) $$\mathrm{respiration}\;\mathrm{rate}\;CO_{2}mg\cdot kg^{-1}\cdot h^{-1}=1.96\times \frac{\left(A_{1}-A_{0}\right)\times \left(V_{1}-V_{0}\right)}{\left(M\times T\right)}$$

The equation for calculating *CO*_2_ concentration is as follows: *A*_0_ represents the *CO*_2_ concentration at 0.5 h, *A*_1_ represents the *CO*_2_ concentration at 1.5 h, *V*_0_ represents the volume of 10 pecan fruits, *V*_1_ represents the volume of the respiration canister, *M* represents the mass of the fruit, and *T* represents the measurement time.

### 2.7. Fruit Quality

The determination of crude fat was carried out using the Soxhlet extractor method [28], the soluble sugar content was determined using the anthrone-sulfuric acid colorimetric method [29], soluble protein was measured using the Coomassie Blue G-250 staining method [30], total phenol content was determined using the Folin reagent method [31].

### 2.8. Statistical Analysis

The experimental data was analyzed using IBM SPSS 22.0 for the analysis of variance (ANOVA), and significant means were separated by Duncan’s test at *p* < 0.05. The correlation was analyzed using Pearson’s correlation analysis. Origin 2018 software was used for graphing.

## 3. Results

### 3.1. Variations in Fruit Quality and Morphology of Pecans

Fruit morphology is an important part of fruit traits and is one of the main factors affecting the quality and commercial value of pecan fruit. It has been shown that there is an obvious interaction effect between pecan fruit morphology and the environment, which will respond differently to different climatic, soil, and other environmental factors, resulting in different introductory results [19]. Therefore, it is of great significance to analyze and study the morphological characteristics of pecan fruit to scientifically evaluate its planting effect. Table 2 presents a summary of the pecans’ green fruit weight, de-greened wet fruit weight, fruit transverse vertical diameter, and fruit shape index at different harvest dates. Both cultivars of pecans exhibited an increasing and then decreasing trend in green fruit weight with the harvest date. There was a significant difference between the weights of the green fruits in ‘Pawnee’ and a non-significant difference between the green fruits in ‘Wichita’. From September 15 to September 20, the weight of green fruits of the ‘Pawnee’ cultivar increased and reached a peak of 42.35 g on September 20. Subsequently, there was a rapid decline in the weight of green fruits from September 20 to October 9, with the lowest point being an average of 30.17 g, significantly lower than the other four harvesting points. The weight of green fruits during the harvesting period ranged from 30.17 g to 42.35 g. The green fruit weight of the ‘Wichita’ cultivar showed an increasing trend from October 9 to October 19, with the highest green fruit weight reaching an average of 29.06 g. The green fruit weight showed a decreasing trend from October 19 to October 29, with an average of 26.07 g on October 29. The weight of green fruit at harvest varied between 23.64 g and 29.06 g. There was no significant variation in the de-greened wet fruit weight of the ‘Pawnee’ cultivar, with an overall trend of decreasing and then increasing. The variation of de-greened wet fruit weight per unit ranged from 13.14 g to 12.45 g. Similarly, there was no significant variation in the de-greened wet fruit weight of the ‘Wichita’ cultivar, and the variation was stabilized between 11.23 g and 12.30 g.

Table 2 shows that the wet fruit unit weights of the ‘Pawnee’ cultivar were higher than those of the ‘Wichita’ cultivar, both when green and de-greened. The maximum green fruit unit weight of the ‘Pawnee’ cultivar was 42.35 g, which was 1.46 times higher than the maximum green fruit unit weight of the ‘Wichita’ cultivar. The ‘Pawnee’ cultivar had a maximum de-greened wet fruit unit weight of 13.14 g, which was 1.07 times higher than the maximum wet fruit unit weight of ‘Wichita’.

Table 2 shows that the transverse and vertical diameters of pecan fruits from the two different cultivars increased as the picking period was delayed. The transverse diameter of the ‘Pawnee’ cultivar fruit was significantly different on September 15 compared to September 25 and October 9, with a maximum of 38.22 mm on October 9. There was no significant difference between September 20 and September 30. The vertical diameter on September 15 was significantly different from that on September 25, reaching a maximum of 65.26 mm, and there were no significant differences between September 20, September 30, and October 9. The transverse diameter of the ‘Wichita’ fruit varied significantly between October 9, 14, and 19, with a maximum of 32.69 mm on October 14. There was no significant difference between October 24 and 29. The longitudinal diameter of the fruit reached a maximum of 57.54 mm on October 24. There was no significant difference in the vertical diameter between October 9 and 29.

The transverse and vertical diameters of pecan fruits for both cultivars exhibited an overall zigzag upward trend. The data on transverse and vertical diameters indicated that the ‘Pawnee’ cultivar had a larger fruit shape than the ‘Wichita’ cultivar. The ‘Pawnee’ fruit had a fuller shape due to a larger increase in transverse diameter in the later stages of development, while the ‘Wichita’ fruit had a narrower and longer shape due to a larger increase in vertical diameter in the later stages of development. The fruit shape index of ‘Pawnee’ varied significantly during the late harvest period, reaching a maximum of 0.59. In contrast, the fruit shape index of ‘Wichita’ remained relatively stable throughout the harvest period.

Table 3 shows the color, fullness, and completeness of pecan seed kernels at different picking periods. As picking was delayed, the seed kernels of ‘Pawnee’ and ‘Wichita’ fruits deepened in color, became more complete, and became less difficult to shell. The ‘Pawnee’ cultivar had fuller seed kernels from 25 September to 30 September, and the ‘Wichita’ cultivar had the fullest seed kernels from October 19 to October 24. 

### 3.2. Variation in Kernel Yield and Moisture Content of Pecan Fruit

**Figure 1:**
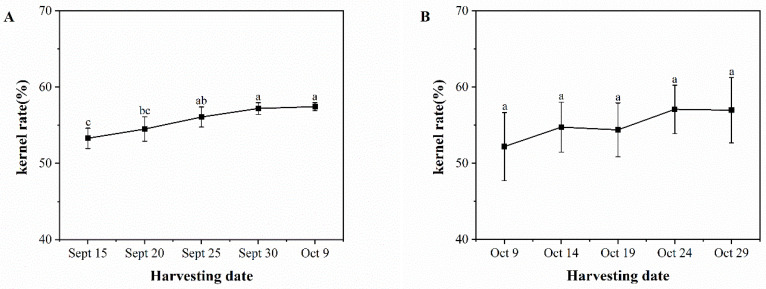
Changes in kernel rates of ‘Pawnee’ (**A**) and ‘Wichita’ (**B**) at different harvesting periods. Means followed by the same letter do not significantly differ at *p* < 0.05.

Figure 1 depicts the variation in kernel yield of ‘Pawnee’ and ‘Wichita’ with sampling dates. The kernel yield of ‘Pawnee’ showed a rapid increase from September 15 to September 30, with less variation from September 30 to October 9. On October 9, the kernel yield reached a maximum value of 57.44%, which was significantly higher than on September 15 (53.29%) and 20 (54.51%). The kernel yield of ‘Wichita’ fruits, in general, showed an increasing trend with no significant difference as the picking date progressed. The lowest kernel yield was 52.19% on October 9, and the highest was 57.08% on October 24.

**Figure 2:**
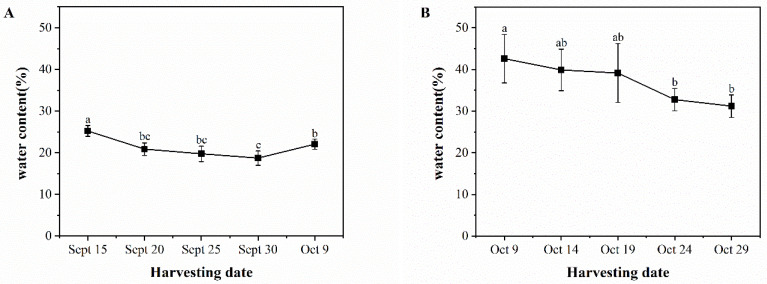
Changes in water content of ‘Pawnee’ (**A**) and ‘Wichita’ (**B**) at different harvesting periods. Means followed by the same letter do not significantly differ at *p* < 0.05.

Figure 2 depicts the variation in moisture content of ‘Pawnee’ and ‘Wichita’ kernels over time. The water content of ‘Pawnee’ kernels showed a decreasing trend, with a greater decrease from September 15 to September 30, followed by a slow increase from September 30 to October 9. This trend may be related to the high rainfall in early October, at the end of the harvest period. On September 15, the maximum water content was 25.22%, which was significantly higher than the values recorded on September 30 (18.72%) and October 9 (22.04%). Figure 2B illustrates the rapid decrease in water content of the ‘Wichita’ seed kernel from October 9 to October 24, followed by a slower decrease from October 24 to October 29. A significant difference was observed between October 9 and October 29, with variations ranging from 31.20% to 42.59%. The kernel yield and water content dynamics showed similar trends for both cultivars. ‘Wichita’ had a higher water content than ‘Pawnee’, but there was little difference in kernel yield.

### 3.3. Color Variations in Pecan Pericarp

**Figure 3:**
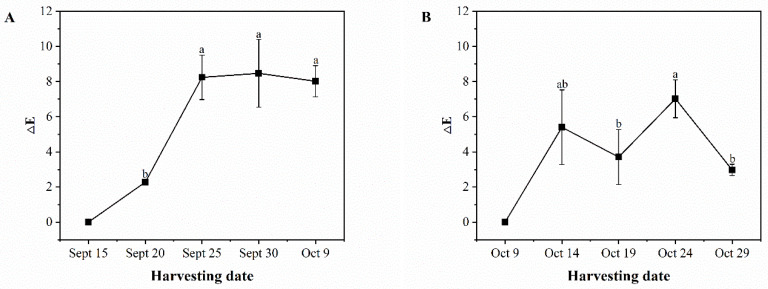
Changes in ∆E (total color difference) of ‘Pawnee’ (**A**) and ‘Wichita’ (**B**) at different harvesting periods. Means followed by the same letter do not significantly differ at *p* < 0.05.

In Figure 3A, it can be observed that the difference in green skin color of ‘Pawnee’ fruits tends to increase and then decrease during the developmental period. The increase was more significant from September 20 to September 25, with ∆E increasing significantly from 2.28 to 8.24. However, from September 25 to October 9, the ∆E values fluctuated slightly. In Figure 3B, it can be observed that the green skin color difference of ‘Wichita’ fruits fluctuated during the developmental period, displaying an increasing and then decreasing trend. The color difference zigzagged from October 9 to October 24, and then decreased sharply from October 24 to October 29, with ∆E decreasing significantly from 7.02 to 2.97.

**Figure 4:**
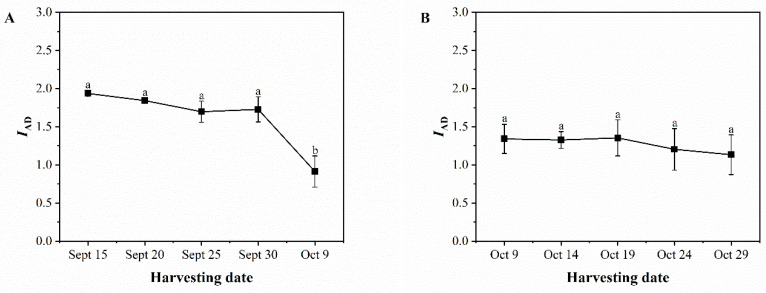
Changes in *I_AD_* (the difference in absorbance at 670 nm and 720 nm) of ‘Pawnee’ (**A**) and ‘Wichita’ (**B**) at different harvesting periods. Means followed by the same letter do not significantly differ at *p* < 0.05.

Figure 4A illustrates a decreasing trend in the *I_AD_* values of the green skin of ‘Pawnee’ fruits. The *I_AD_* values were smaller in more mature fruits, with significantly lower values on October 9 (0.91) than on September 15 (1.94). Figure 4B demonstrates that the *I_AD_* value of the green skin of the ‘Wichita’ fruit remained relatively stable, at around 1.3, from October 9 to October 19. It then dropped to 1.13 from October 24 to October 29.

### 3.4. Variation in Respiration Intensity of Pecan Fruit

**Figure 5:**
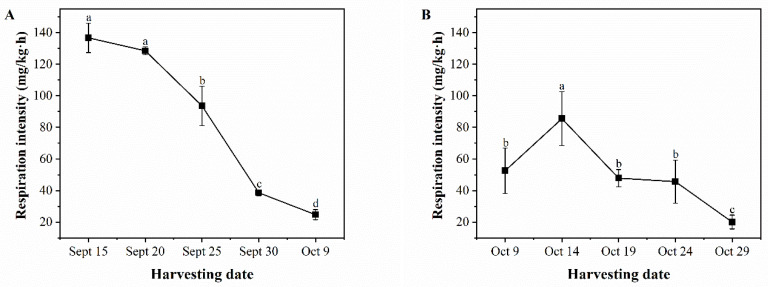
Changes in respiratory intensity of ‘Pawnee’ (**A**) and ‘Wichita’ (**B**) at different harvesting periods. Means followed by the same letter do not significantly differ at *p* < 0.05.

In Figure 5A, it can be observed that the respiration intensity of ‘Pawnee’ fruits decreased from September 15 to October 9. The respiration intensity during the late harvest was significantly lower than that during the pre-harvest stage, with a variation range of 136.70 mg/kg·h to 24.72 mg/kg·h. Figure 5B shows an increasing and then decreasing trend in the respiration intensity of ‘Wichita’ fruits from October 9 to October 29. The respiratory intensity reached a maximum of 85.52 mg/kg·h on October 14 and dropped to a minimum of 20.06 mg/kg·h. The respiration intensity of pecan fruit decreases as the harvesting period is delayed, indicating that the fruit’s ability to withstand adverse external environments gradually strengthens, resulting in better storage properties [32].

### 3.5. Crude Fat

**Figure 6:**
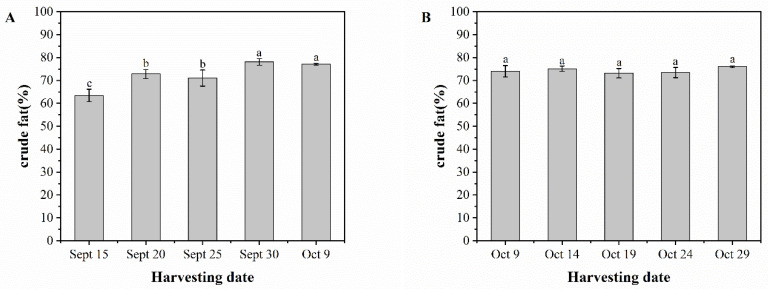
Crude fat content of ‘Pawnee’ (**A**) and ‘Wichita’ (**B**) at different harvesting periods. Means followed by the same letter do not significantly differ at *p* < 0.05.

Tests were conducted to determine the crude fat content of two varieties of pecan fruit. The results showed an increasing trend, with the highest crude fat content being observed in the later stages of fruit development. Figure 6A illustrates that the crude fat content of ‘Pawnee’ fruits peaked at 78.10% on September 30. There was a highly significant difference between the crude fat content of fruits on September 15 (63.45%) and that of fruits on October 9 (77.07%). The crude fat content of fruits in the late harvest period increased by about 21.5% compared to that of fruits in the early harvest period. The crude fat content of ‘Wichita’ fruits varied more gently without significant differences, with the lowest crude fat content of 73.20% in fruits on October 19 and the highest crude fat content of 75.99% in fruits on October 29, representing an increase of approximately 3.8%. ‘Pawnee’ fruits had a lower crude fat content than ‘Wichita’ fruits during the pre-developmental period. Additionally, the growth of crude fat content was slightly higher in ‘Pawnee’ fruits than in ‘Wichita’ fruits during the post-developmental period.

### 3.6. Soluble Sugar

**Figure 7:**
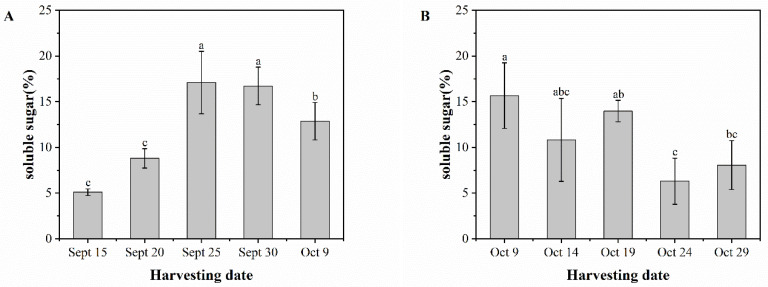
Soluble sugar content of ‘Pawnee’ (**A**) and ‘Wichita’ (**B**) at different harvesting periods. Means followed by the same letter do not significantly differ at *p* < 0.05.

Figure 7A illustrates that the soluble sugar content of ‘Pawnee’ fruits showed an increasing and then decreasing trend with the harvest period. From September 15 to September 25, the soluble sugar content increased extremely significantly from 5.10% to 17.10%, which was an increase of about 235.3%. However, from September 25 to October 9, the soluble sugar content decreased significantly from 17.10% to 12.87%, a decrease of about 32.9%. The soluble sugar content of ‘Wichita’ fruits decreased from 15.67% on October 9 to 10.83% on October 14. It then increased to 13.97% on October 19 before declining significantly and leveling off at 6.30% to 8.07% between October 24 and October 29. Similarly, the soluble sugar content of the two cultivars of pecan fruits varied in the pre-harvest period, but both showed a decreasing trend in the post-harvest period.

### 3.7. Soluble Protein

**Figure 8:**
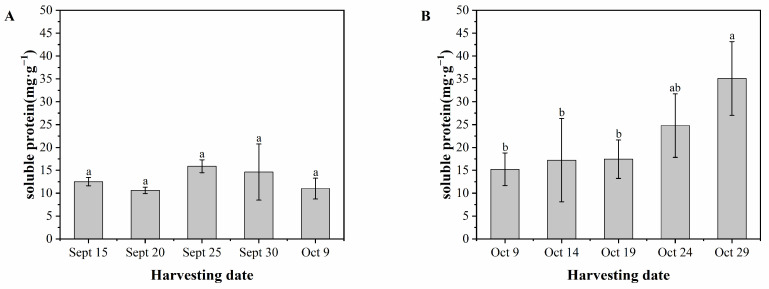
Soluble protein content of ‘Pawnee’ (**A**) and ‘Wichita’ (**B**) at different harvesting periods. Means followed by the same letter do not significantly differ at *p* < 0.05.

Figure 8A indicates that there was no significant difference in the soluble protein content of ‘Pawnee’ fruits during the harvest period. The mean values of soluble protein content for the five harvest dates from September 15 to October 9 were 12.53 mg·g^−1^, 10.62 mg·g^−1^, 15.89 mg·g^−1^, 14.62 mg·g^−1^, and 11.01 mg·g^−1^, respectively. Figure 8B shows that the soluble protein content of ‘Wichita’ fruits increased over time. The content remained stable from October 9 to October 19 and then significantly increased from October 19 to October 29. The lowest soluble protein content was 15.23 mg·g^−1^ on October 9, while the highest was 35.08 mg·g^−1^ on October 29. Overall, ‘Wichita’ fruits had higher soluble protein content than ‘Pawnee’ fruits.

### 3.8. Total Phenol

**Figure 9:**
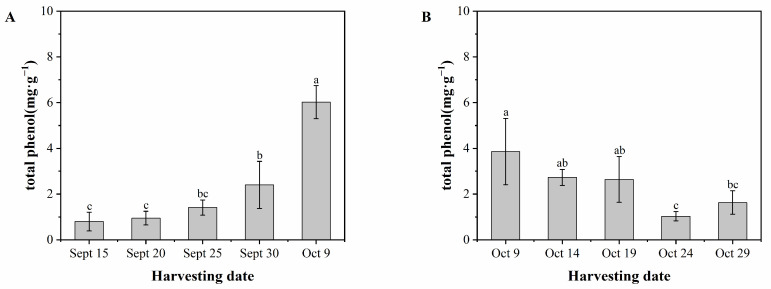
Total phenol content of ‘Pawnee’ (**A**) and ‘Wichita’ (**B**) at different harvesting periods. Means followed by the same letter do not significantly differ at *p* < 0.05.

The total phenolic content of ‘Pawnee’ fruits increased with the delay in the picking period. Figure 9A shows a slow increase in total phenolic content from September 15 (0.80 mg·g^−1^) to September 30 (2.40 mg·g^−1^), followed by a rapid increase to the highest value from September 30 (2.40 mg·g^−1^) to October 9 (6.02 mg·g^−1^), with a growth rate of 150.8%. The total phenol content of ‘Wichita’ fruits exhibited a decreasing trend initially, followed by an increasing trend with the picking period. There were significant differences in total phenols between October 9 and October 24 fruits, with the highest being 3.86 mg·g^−1^ and the lowest being 1.03 mg·g^−1^. The total phenols increased to 1.63 mg·g^−1^ on October 29. When comparing the post-harvest samples of the two cultivars, the total phenolic content of ‘Pawnee’ fruits was approximately 269.3% higher than that of ‘Wichita’.

### 3.9. Correlation Analysis

Table 4 analyses the correlation between phenotypic characteristics and nutrients of the two pecan cultivars, showing highly significant positive correlations between green fruit weight and de-greened wet fruit weight, transverse vertical diameter, respiration intensity, and *I_AD_*, with the highest correlation index between green fruit weight and fruit vertical diameter at 0.793**. There was a highly significant positive correlation between de-greened wet fruit weight, and transverse vertical diameter, with the highest correlation index of 0.706** with vertical diameter. The water content demonstrated a highly significant negative correlation with green fruit weight, de-greened wet fruit weight and transverse vertical diameter, with the strongest correlation observed between water content and vertical diameter (−0.788**). The transverse diameter demonstrated a highly significant positive correlation with green fruit weight, de-greened wet fruit weight, vertical diameter, and ΔE, and the correlation index was the highest for vertical diameter, at 0.845**. The vertical diameter exhibited a highly significant negative correlation with water content and fruit shape index. A highly significant positive correlation was identified between respiratory intensity, green fruit weight, and *I_AD_*, with a maximum correlation index of 0.731**. Additionally, a highly significant negative correlation was observed between respiratory intensity and crude fat and total phenols, with a maximum negative correlation index of −0.705** for respiratory intensity and crude fat. A highly significant positive correlation was demonstrated between ∆E and kernel rate (0.551**) and transverse diameter (0.559**). A highly significant negative correlation was observed between total phenols and respiration intensity (−0.471**) and *I_AD_* (−0.524**). The results demonstrated a strong correlation between fruit weight, fruit transverse and vertical diameter, and water content. Additionally, a significant relationship was observed between fruit respiratory intensity, crude fat, and total phenols.

## 4. Discussion

The initial observation of pecan maturity is discernible through the outer pericarp, which exhibits a bright green hue and subsequently transitions to a yellowish-green as it ripens. Should harvesting remain postponed, the outer pericarp will progressively assume a crumpled appearance and develop dark brown spots. The color difference ΔE value represents the difference in peel color change [33]. A larger value of ΔE represents a greater degree of color change. The *I_AD_* values are calculated based on changes in chlorophyll content in the pericarp to reflect fruit maturity. The *I_AD_* value is determined by the difference in absorbance at 670 nm and 720 nm of the pericarp, and the interval ranges from 0 to 2.2. A smaller measured value indicates a higher level of ripeness in the fruit [26,27]. In this study, it was found that the color difference of the green peel of ‘Pawnee’ fruits fluctuated more than that of ‘Wichita’. However, both peel *I_AD_* values decreased gradually with the delay in harvesting. This study investigated the quality of pecan fruit at different harvesting periods, focusing on external phenotypic characteristics and intrinsic nutrients. External phenotypic characteristics examined were fruit mass, transverse and longitudinal diameters, fruit shape index, seed kernel color, seed kernel fullness, kernel emergence rate, and water content. Intrinsic nutrients analyzed were crude fat, soluble sugars, soluble proteins, and total phenols.

Product quality is a crucial economic indicator for evaluating crop production. The quality of the product is closely related to cultivation management, variety selection, and timely harvesting. The study demonstrated that as the pecan fruit matured gradually and the picking time was delayed, the seed kernel became full and turned golden yellow. Additionally, the green fruit weight of both ‘Pawnee’ and ‘Wichita’ showed an increasing trend followed by a decreasing trend. This indicates that the quality of the green fruits steadily improved as the fruits developed. Furthermore, the green skins split and gradually lost water, resulting in a decrease in the quality of the green fruits as they matured. Both cultivars of pecan exhibited an overall zigzag upward trend in fruit transverse vertical diameter. Significant changes in transverse vertical diameter were observed in the later stages of fruit picking compared to the earlier stages. The ‘Pawnee’ cultivar had a greater transverse vertical diameter, green fruit weight, and wet fruit weight than the ‘Wichita’ cultivar. The moisture content had a negative correlation with the kernel yield of pecan fruit. There was a general decrease in moisture content, and the highest kernel yields were observed towards the end of the harvest. 

There are significant differences in the nutrients of pecans at different harvest dates, which are a key measure of fruit quality. The experimental results indicate that the crude fat content of the fruits exhibited a slight fluctuation but gradually increased with the delay of the harvesting period. This finding is consistent with the results of previous studies by Wu Jing [34] and Jia Xiaodong [35]. The soluble sugar content of ‘Pawnee’ fruits exhibited an initial increase, followed by a decrease over the harvesting period. The ‘Wichita’ fruit soluble sugar change curve had a ‘W’ shape. Soluble sugar accumulates during the early stages of harvest to provide enough vitality for the young embryo of the pecan seed kernel. As the fruit ripened, the soluble sugar content decreased, and the sugar substances in the seed kernel were gradually converted to fat. During the late harvest period, the soluble sugar content of ‘Wichita’ fruits leveled off and tended to increase. This may be attributed to the fact that at this stage, the embryo is fully mature, the water content of the seed kernel decreases, and the intensity of respiration is lowered, resulting in a significant reduction in nutrient consumption by the fruit. The change curves for soluble protein and soluble sugar in ‘Pawnee’ fruits were similar. Both showed an increasing trend, followed by a decrease as the harvesting period was delayed. This is consistent with the findings of Chang Jun [36] and Jia Xiaodong [35]. As the seed kernels of ‘Pawnee’ fruit mature, soluble sugars and soluble proteins are gradually converted to crude fat, resulting in a significant increase in crude fat content compared to the early harvesting period. The soluble protein in ‘Wichita’ fruit exhibited an increasing trend, consistent with the findings of Hao Jinlian [37] and Wang Yiying [13], who reported variations in inclusion trends among different pecan cultivars. The underlying reasons for the differing inclusion trends in ‘Pawnee’ and ‘Wichita’ fruits require further investigation. The above results indicate that delaying the harvesting period appropriately according to the local climatic conditions is beneficial for the material accumulation of pecan kernels, leading to an increase in economic yield. This finding is consistent with previous reports [12,13,36,38,39]. The optimal harvesting period for pecans also coincides with the time when the phenotypic characteristics and quality of de-greened wet fruit are at their peak. This study contributes to the refinement of our understanding of pecan ripening performance. The optimal harvest time can be used as a reference point for other regions with similar geographic and environmental conditions. In regions with significant differences in geographic and climatic conditions, the data can be used as a basis for side-by-side comparisons to understand the differences in pecan cultivation performance and harvesting in different regions of China. This will provide fruit growers and distributors with a more comprehensive set of information to inform their production decisions.

## 5. Conclusions

This study analyzed the phenotypic characteristics and quality of two pecan cultivars, ‘Pawnee’ and ‘Wichita’, in Nanjing at different harvesting periods. The results indicate that appropriately delaying the harvesting during the maturity period of pecans can improve the yield and quality of the fruits. In production, farmers start harvesting the fruit when the natural cracking of the skin reaches about 30 percent of the total fruit. Harvesting machinery shakes the branches, and some of the unripe fruit is shaken off. A common problem is that the fruit is harvested too early and not according to variety. Pecans harvested prematurely exhibit characteristics of immaturity, including smaller fruits, lower kernel yield, incomplete cracking of the green skin, and a tendency to hinder the accumulation of seed kernel fat. These factors collectively affect the commercial value of pecans. A comparison of the single fruit quality, fruit transverse and vertical diameter, fruit shape index, seed kernel color, seed kernel fullness, kernel rate, water content, respiration intensity, crude fat, soluble sugar, soluble protein, and total phenols, optimum harvesting period for the pecan ‘Pawnee’ cultivar in the Nanjing area is from the end of September to the beginning of October, and that for the pecan ‘Wichita’ cultivar is from the mid-end to the end of October in the Nanjing area.

## Figures and Tables

**Table 1:** Ripening time of the main pecan varieties grown in different provinces.

Cultivar	Provinces	Geographic Location	Climatic Characteristics	Frost-Free Period	Ripening Period
Jinhua 1	Yunnan [12]	97°31′~106°11′ E, 21°8′~29°15′ N	The climate is semi-humid, situated in the central subtropical zone. The mean annual temperature is 16.5 °C, with an average annual precipitation of 1056 mm.	301 d	Mid-October to early November
Pawnee	Hunan [13]	110°59′~110°40′ E, 26°40′~27°6′ N	It has a subtropical monsoon climate with an average annual temperature of 16.6 °C and an average annual precipitation of 1491 mm.	290 d	Around 20 October
Mahan					
Pawnee	Shandong [14]	115°16′~116°32′ E, 35°47′~37°02′ N	The region exhibits a temperate monsoon climate, with an average annual temperature of 13.5 °C and an average annual precipitation of 540.4 mm.	208 d	Mid-October to early November
Mahan					Mid-late October to early November
Mahan	Zhejiang [15]	118°01′~123°10′ E, 27°02′~31°11′ N	The region has a subtropical monsoon climate with an average annual temperature of 17.3 °C and an average annual precipitation of 1406 mm.	257 d	Around October
Pawnee	Guangxi [16]	107°5′~107°43′ E, 24°13′~24°51′ N	The region has a subtropical monsoon climate with an average annual temperature of 19.9 °C and an average annual precipitation of 1389.1 mm.	290 d	Mid to late September
Shawnee					Late September to early October
Mahan					Mid to late October
caddo					Early to mid October
Pawnee	Jiangxi [17]	113°34′~118°28′ E, 24°29′~30°04′ N	The region has a subtropical, warm, and humid monsoon climate, with an average annual temperature of 19.1 °C and an average annual precipitation of 1576.3 mm.	214 d	Mid-September
Wichita					Mid-October
Shawnee					Early to mid-October
Chengyin 15	Gansu [18]	104°01′~106°35′ E, 32°35′~34°32′ N	The region has a warm temperate continental climate with an average annual temperature of 11.9 °C and an average annual precipitation of 639 mm.	210 d	Between 20 September and 2 October
Xinyin 5	Henan [19]	110°21′~116°39′ E, 31°23′~36°22′ N	The region has a subtropical warm and humid climate with an average annual temperature of 15.3 °C and an average annual rainfall of 1109.1 mm.	221 d	September to October

**Table 2:** Fruit morphology of the two cultivars at different harvesting periods.

Cultivar	Harvest Dates	Green Fruit Weight/g	De-Greened Wet Fruit Weight g	Transverse Diameter (mm)	Vertical Diameter (mm)	Fruit Shape Index
Pawnee	September 15	41.64 ± 2.51 a	13.14 ± 0.89 a	34.20 ± 1.03 b	61.20 ± 2.02 b	0.56 ± 0.03 ab
	September 20	42.35 ± 3.69 a	13.08 ± 0.56 a	35.05 ± 2.10 ab	63.73 ± 2.57 ab	0.55 ± 0.01 b
	September 25	41.26 ± 2.29 a	12.45 ± 0.65 a	38.05 ± 1.00 a	65.26 ± 1.52 a	0.59 ± 0.01 a
	September 30	35.88 ± 2.47 b	12.76 ± 0.46 a	36.50 ± 0.22 ab	63.24 ± 0.53 ab	0.58 ± 0.01 ab
	October 9	30.17 ± 3.16 c	13.05 ± 0.61 a	38.22 ± 2.67 a	64.74 ± 2.29 ab	0.59 ± 0.02 a
Wichita	October 9	23.64 ± 3.86 a	11.23 ± 1.14 a	30.95 ± 1.14 b	51.98 ± 4.10 a	0.60 ± 0.03 a
	October 14	25.26 ± 3.81 a	12.07 ± 1.09 a	32.69 ± 1.05 a	56.49 ± 5.98 a	0.58 ± 0.05 a
	October 19	29.06 ± 3.94 a	12.30 ± 0.68 a	32.61 ± 0.42 a	56.99 ± 1.41 a	0.57 ± 0.01 a
	October 24	27.07 ± 3.95 a	11.29 ± 0.70 a	31.66 ± 0.65 ab	57.54 ± 5.86 a	0.55 ± 0.05 a
	October 29	26.07 ± 4.61 a	12.09 ± 1.19 a	32.48 ± 0.69 ab	56.84 ± 5.10 a	0.57 ± 0.04 a

Means followed by the same letter do not significantly differ at *p* < 0.05. Data are mean values (*n* = 15) ± SD.

**Table 3:** Comparison of seed kernels at different harvesting periods.

Cultivar	Harvest Dates	Kernel Color	Fullness	Intactness
Pawnee	September 15	Light yellow	Empty	≥1/2
	September 20	Light yellow	Fuller	≥1/2
	September 25	Golden yellow, partly dark brown	fullness	≥1/2
	September 30	Golden yellow, partly dark brown	fullness	≥1/2
	October 9	Dark brown	Shrinkage	≥1/2
Wichita	October 9	Light yellow	Empty	≥1/2
	October 14	Light yellow	Fuller	≥1/2
	October 19	Light yellow	fullness	≥1/2
	October 24	Golden yellow, partly light yellow	fullness	≥1/2
	October 29	Golden yellow, partly dark brown	Fullness, partly shrinkage	≥1/2

**Table 4:** Correlation analysis between phenotypic characters and nutrients in two cultivars of pecan.

	Green Fruit Weight	De-Greened Wet Fruit Weight	Kernel Rate	Water Content	Transverse Diameter	Vertical Diameter	Fruit Shape Index	Respiratory Intensity	∆E	*I_AD_*	Soluble Sugar	Crude Fat	Soluble Protein	Total Phenol
Green fruit weight	1													
De-greened wet fruit weight	0.676 **	1												
Kernel rate	0.064	−0.102	1											
Water content	−0.744 **	−0.500 **	−0.454 *	1										
Transverse diameter	0.652 **	0.578 **	0.314	−0.761 **	1									
Vertical diameter	0.793 **	0.706 **	0.309	−0.788 **	0.845 **	1								
Fruit shape index	−0.409 *	−0.378 *	−0.080	0.225	0.049	−0.491 **	1							
Respiratory intensity	0.600 **	0.214	−0.341	−0.181	0.073	0.141	−0.117	1						
∆E	0.067	0.070	0.551 **	−0.422	0.559 *	0.420 *	0.122	−0.378 *	1					
*I_AD_*	0.692 **	0.264	−0.061	−0.430 *	0.186	0.248	−0.150	0.731 **	−0.233	1				
Soluble sugar	0.016	0.025	−0.056	−0.017	0.362 *	0.101	0.397 *	−0.246	0.353	−0.019	1			
Crude fat	−0.446 *	−0.152	0.422 *	−0.013	0.038	-0.028	0.103	−0.705 **	0.437 *	−0.418 *	0.310	1		
Soluble protein	−0.446 *	−0.330	0.112	0.337	−0.424 *	−0.398 *	0.061	−0.389 *	−0.027	−0.340	−0.209	0.199	1	
Total phenol	−0.446 *	−0.115	0.101	0.166	0.168	−0.104	0.459 *	−0.471 **	0.201	−0.524 **	0.397 *	0.450 *	−0.239	1

Note: “**” Correlation is significant at the 0.01 level, “*” Correlation is significant at the 0.05 level.

## Data Availability

The original contributions presented in the study are included in the article, further inquiries can be directed to the corresponding author.

## References

[1] Sparks D. (2005). Adaptability of Pecan as a Species. HortScience.

[2] Jia X., Wang T., Zhang J., Wang X., Liu Y., Guo Z. (2012). A Review of Progress in *Caryaillinoensis*. Chin. Agric. Sci. Bull..

[3] Li Y., Le D. (2015). *Carya illinoensis* fruit forest industry development thinking and cultivation key technology. J. For. Eng..

[4] Domínguez-Avila J.A., Alvarez-Parrilla E., López-Díaz J.A., Maldonado-Mendoza I.E., Gómez-García M.D.C., De La Rosa L.A. (2015). The Pecan Nut (*Carya Illinoinensis*) and Its Oil and Polyphenolic Fractions Differentially Modulate Lipid Metabolism and the Antioxidant Enzyme Activities in Rats Fed High-Fat Diets. Food Chem..

[5] Cao F., Tan P., Peng F. (2017). Research Progress of Vegetative Propagation Technology for Pecan (*Carya illinoinensis*). World For. Res..

[6] Wu X., Beecher G.R., Holden J.M., Haytowitz D.B., Gebhardt S.E., Prior R.L. (2004). Lipophilic and Hydrophilic Antioxidant Capacities of Common Foods in the United States. J. Agric. Food Chem..

[7] Vadivel V., Kunyanga C.N., Biesalski H.K. (2012). Health Benefits of Nut Consumption with Special Reference to Body Weight Control. Nutrition.

[8] Xu H., Cheng H., Wang Z., Fu S., Si J., Yu M., Zhang A. (2016). The Study of Total Polyphenols, Total Flavonoids and Antioxidant Capacity in Pecan [*Carya illinoinensis* (Wangenh.) K. Koch] Kernerls. J. Nucl. Agric. Sci..

[9] Robbins K.S., Greenspan P., Pegg R.B. (2016). Effect of Pecan Phenolics on the Release of Nitric Oxide from Murine RAW 264.7 Macrophage Cells. Food Chem..

[10] Zhang R., Peng F., Li Y. (2015). Pecan Production in China. Sci. Hortic..

[11] Zhong L., Dong X. (2018). Development Status and Countermeasures of *Carya illinoensis* and Oleaginous Peony in Jiangsu. J. Jiangsu For. Sci. Technol..

[12] Dong R. (2002). Study of Fruit Development of *Carya illinoensis*. J. West. China For. Sci..

[13] Wang Y., Zhou W., Lu K., Zeng S., Yuan J. (2022). Fruit morphology and quality variation patterns of four *Carya illinoinensis* varieties in Hunan province. Nonwood For. Res..

[14] Tang G., Zhao D., Liu B., Zhang X., Yao Y., Cui P., Ma Y. (2020). Introduction of several fine cultivars of *Carya illinoensis* suitable for cultivation in Shandong. J. Shandong For. Sci. Technol..

[15] Chang J., Ren H., Yao X., Yang S., Zhang S., Zhang C., Wang K. (2021). A Comparative Analysis of Nutritional Components and Fatty Acid Composition of 41 Pecan Varieties. J. Southwest Univ. (Nat. Sci.).

[16] Deng L., Pan H., Ma T., Wei Z., Wang F., Qin F., Wei L., Wen J., Tan Y., Ou J. (2022). Growing performance and quality characteristics of six thin-shelled pecan varieties planted in Hechi city of Guangxi. Nonwood For. Res..

[17] Zuo J., He Y., Huang J., Zhai M. (2023). Main Cultivars and High Yield Cultivation Techniques of *Carya illinoensis* in Jiangxi. Contemp. Hortic..

[18] Zhu F., Guo X., Hu J., Zhang K. (2015). Preliminary Study on Introduction Experiment of *Carya illinoensis*. Gansu Agric..

[19] Zhang J., Shen M., Zhang L., Zhou Y., Yu K., Zhou C., Gao C. (2022). Analysis of Fruit Morphological Characteristics of Different *Carya illinoensis*. Shanxi J. Agric. Sci..

[20] Zhang R., Lv F. (2002). Pecan Distribution, Cultural Regionalization and Cultivar Classification in USA. Nonwood For. Res..

[21] Xi X., Fan Z., Zou W., Liao Y., Dong R. (2006). Introduction of ten *Carya illionoensis* cultivars. J. Zhejiang A F Univ..

[22] Fang L., Wu W., Li Y., Liu Y., Zhai M., Li X. (2010). Study on fruit quality of different cultivars of *Carya illinoensis* planted in Nanjing area. Jiangsu Agric. Sci..

[23] Sheng J., Zang X., Zhou B., Zhu H., Liu G. (2012). *Carya illinoinensis* cultivars and cultivation technology suitable for Jiangsu planting. Jiangsu Agric. Sci..

[24] Filho A.C., Poletto T., Muniz M.F.B., Baggiotto C., Poletto I., Filho A.C., Poletto T., Muniz M.F.B., Baggiotto C., Poletto I. (2015). Sample Size for Evaluating the Weight and Diameter of Pecan Fruits. Ciênc Rural.

[25] (2016). Quality Grade of Pecan Nuts and Kernels.

[26] Ziosi V., Noferini M., Fiori G., Tadiello A., Trainotti L., Casadoro G., Costa G. (2008). A New Index Based on Vis Spectroscopy to Characterize the Progression of Ripening in Peach Fruit. Postharvest Biol. Technol..

[27] Farneti B., Gutierrez M.S., Novak B., Busatto N., Ravaglia D., Spinelli F., Costa G. (2015). Use of the Index of Absorbance Difference (IAD) as a Tool for Tailoring Post-Harvest 1-MCP Application to Control Apple Superficial Scald. Sci. Hortic..

[28] Singanusong R., Mason R.L., D’Arcy B.R., Nottingham S.M. (2003). Compositional Changes of Australia-Grown Western Schley Pecans [*Carya Illinoinensis* (Wangenh.) K. Koch] during Maturation. J. Agric. Food Chem..

[29] Mo S., Qian C. (1992). Colorimetric determination of soluble sugars in fruits. J. Fruit Sci..

[30] Qu C., Shen S., Wang X., Cui Y., Song W. (2006). Method research of measuring soluble protein contents of plant rough extraction using Coomassie Brilliant Blue. J. Suzhou Univ. Nat. Sci. Ed..

[31] Siano F., Moccia S., Picariello G., Russo G., Sorrentino G., Di Stasio M., La Cara F., Volpe M. (2018). Comparative Study of Chemical, Biochemical Characteristic and ATR-FTIR Analysis of Seeds, Oil and Flour of the Edible Fedora Cultivar Hemp (*Cannabis Sativa* L.). Molecules.

[32] Gong B., Jiang L., Ma H., Wang J. (2014). Effect of Harvest Date on Cold Storage and Postharvest Physiology of Green Walnut Fruits. Food Sci..

[33] Gao R., Hu Y., Yang Y., Gong F., Wang W., Wang H., Wang X., Chen X., Xu L., Li L. (2021). Dynamic changes of major physicochemical characteristics of Longnan “Jinlong 2” walnut with maturity. China Oils Fats.

[34] Wu J. (2018). The effects of different harvest time on the quality of walnut fruits. J. Shanxi. Agric. Univ. (Nat. Sci.).

[35] Jia X., Luo H., Zhai M., Qian M., Liu Y., Li Y., Guo Z., Qiao Y. (2016). Dynamic changes and correlation analysis of nutrient contents in ‘Pawnee’ pecan (*Carya illinoinensis*). J. Fruit Sci..

[36] Chang J., Ren H., Yao X., Yang S., Wang K., Zhang C. (2019). Dynamic development and nutrient accumulation regularity in Carya illinoensis ‘Mahan’ nut. Nonwood For. Res..

[37] Hao J., Wang R., Luo S., Chen H., Hu H. (2023). Preliminary Study on Effect of Harvesting Times on the Quality of Six Walnut Varieties in Yecheng County. Nucl. Agric. Sci..

[38] Jia X., Luo H., Zhai M., Li Y., Guo Z., Qiao Y. (2015). Dynamic analysis of pecan (*Carya illinoensis* ‘Pawnee’) nut development. J. Fruit Sci..

[39] Chen W., Liu X., Deng Q., Peng F., He H., Li X. (2016). Nut development and fatty acid accumulation in *Carya illinoensis*. Nonwood For. Res..

